# Effectiveness of Empower-Grief for Relatives of Palliative Care Patients: Protocol for an Exploratory Randomized Controlled Trial

**DOI:** 10.32872/cpe.14307

**Published:** 2025-02-28

**Authors:** David D. Neto, Alexandra Coelho, Sara Albuquerque, Ana Nunes da Silva

**Affiliations:** 1 School of Psychology, ISPA – Instituto Universitário, Lisbon, Portugal; 2 APPsyCI – Applied Psychology Research Center Capabilities & Inclusion, Lisbon, Portugal; 3 HEI‐Lab: Digital Human‐Environment Interaction Labs, Lusófona University, Lisbon, Portugal; 4 Faculdade de Psicologia, Universidade de Lisboa, Lisbon, Portugal; 5 CICPSI, Lisbon, Portugal; Philipps-University of Marburg, Marburg, Germany

**Keywords:** prolonged grief disorder, palliative care, Empower-Grief, psychological intervention, bereavement

## Abstract

**Background:**

Grief reactions of relatives of palliative care patients are seldom addressed. Most interventions focus on Prolonged Grief Disorder (PGD) and not on its prevention. This is particularly relevant in palliative care, in which death is the result of a difficult period of a terminal illness, making caregivers particularly vulnerable to psychological distress. The purpose of the present exploratory trial is to test the efficacy of a selective intervention (Empower-Grief) for the initial problematic grief reactions and to study potential predictors of adherence and efficacy.

**Method:**

This is an exploratory Randomized Controlled Trial (RCT) studying Empower-Grief compared with Treatment as Usual (TAU). Participants will be relatives or caregivers of palliative and oncological patients with initial indicators of risk of developing PGD and will be randomly allocated to Empower-Grief and TAU. Participants will be assessed prior, at the end and six months after the intervention. The primary outcome considered will be symptoms of PGD. The assessment includes measures of anxiety and depression, coping, attachment, psychological flexibility, posttraumatic growth, social support and therapeutic alliance.

**Results:**

The trial is ongoing. Forty-four participants will be invited to participate.

**Conclusion:**

This study addresses the need for the development of empirically grounded and feasible interventions aimed at dealing with initial problematic reactions in grief, exploring potential predictors and possible venues for personalizing intervention and understanding the mechanism through which these interventions operate.

## Highlights

- Caregivers of deceased cancer patients are particularly vulnerable to Prolonged Grief Disorder.
- There is a significant need to develop low-intensity interventions for emotional reactions to grief.
- The current protocol aims to test the efficacy of Empower-Grief and to identify predictors of outcome.

## Background

Prolonged Grief Disorder (PGD), recognized as a mental illness in the ICD-11 (World Health Organization, 2019) and more recently in the DSM-5-TR (American Psychiatric Association, 2022), consists of intense grief along with significant social and occupational dysfunction persisting for an extended period (with the inclusion in the DSM-5-TR, the temporal criterion was extended from 6 to 12 months).

Caregivers of patients with advanced cancer are particularly vulnerable to elevated psychological distress. In a caregiver sample, PGD prevalence was 40% at six months, 28% at 13 months, and 27% at 18 months (Guldin et al., 2012), thus supporting the idea that end-of-life caregiving increases the risk of mental health disturbance. High levels of PGD were also found in a Portuguese population of palliative care caregivers (Coelho et al., 2022).

Considering the elevated prevalence of disordered reactions, the goal of providing different interventions according to the risk of developing PGD has the potential to provide care to more individuals. These interventions range from psychoeducation (i.e., universal interventions) to low-intensity selective and high-intensity indicative interventions. Very few low-intensity interventions have been proposed. Lichtenthal and colleagues (Lichtenthal et al., 2022) developed a brief manualized cognitive-behavioural, acceptance-based intervention for critically ill patients´ caregivers called EMPOWER (Enhancing and Mobilizing the POtential for Wellness and Resilience). EMPOWER is a psychological intervention composed of six modules based on cognitive-behavioural and acceptance-based interventions: 1) initial assessment, rationale and adherence to the intervention; 2) resources for stabilization; 3) psychoeducation on grief and the cognitive-behavioural model; 4) promoting experiential acceptance; 5) imagined dialogue; 6) coping training. This intervention has been shown to be feasible, acceptable and effective in reducing psychological symptoms, including PGD, depression and anxiety. This program, designed initially for intensive care contexts, emphasizes the detrimental role of experiential avoidance (i.e., the tendency to avoid unpleasant feelings) in decision-making and the grieving process. As opposed to acceptance, this coping mechanism has been associated with higher anticipatory grief (Davis et al., 2017) and the persistence of complicated grief in palliative care (Eisma & Stroebe, 2021).

The majority of the family caregivers will require selective intervention, oriented at people identified as being at risk of developing PGD. The differentiation of interventions allows combining need-based timely interventions to prevent PGD while increasing access through rationalization of service delivery. Despite the recommendations, no studies have matched the intensity of interventions to the severity of psychological reaction to death in preventing prolonged grief. The present research intends to implement and evaluate the effectiveness of the Empower-Grief intervention (Coelho et al., 2024; Lichtenthal et al., 2022) in the population of family caregivers of patients accompanied in the Palliative Medicine Unit of the Centro Hospitalar Universitário Lisboa Norte (CHULN). Empower-Grief is an adaptation of the original EMPOWER to the current palliative care context and post-mortem stage. It consists of the same six modules of the original intervention, but a 50-minute session is devoted to each of the modules, with two booster sessions done after treatment. It is a manualized treatment in which each session is structured.

This research project has two objectives. First, to evaluate the effectiveness of Empower-Grief Intervention compared to treatment as usual (TAU) in terms of symptoms of prolonged grief and psychological distress in relatives or caregivers of palliative and oncological patients. The second objective is to identify predictors, among the factors consistently found in the literature, of response to intervention. This second objective is crucial, considering the low-intensity level of the intervention. In addition to the risk of prolonged grief, other factors such as prior mental health, the nature of the death, social support, attachment style and psychological flexibility may be relevant in explaining the response to this intervention and serve as essential variables in adapting the intervention to the needs of the participants. This research is crucial to influence national policy towards a greater emphasis on prevention and early intervention, making the allocation of cost-effective bereavement support services the most efficient and sustainable approach for a significant public health impact in bereavement care.

## Method

### Design

**Figure 1:**
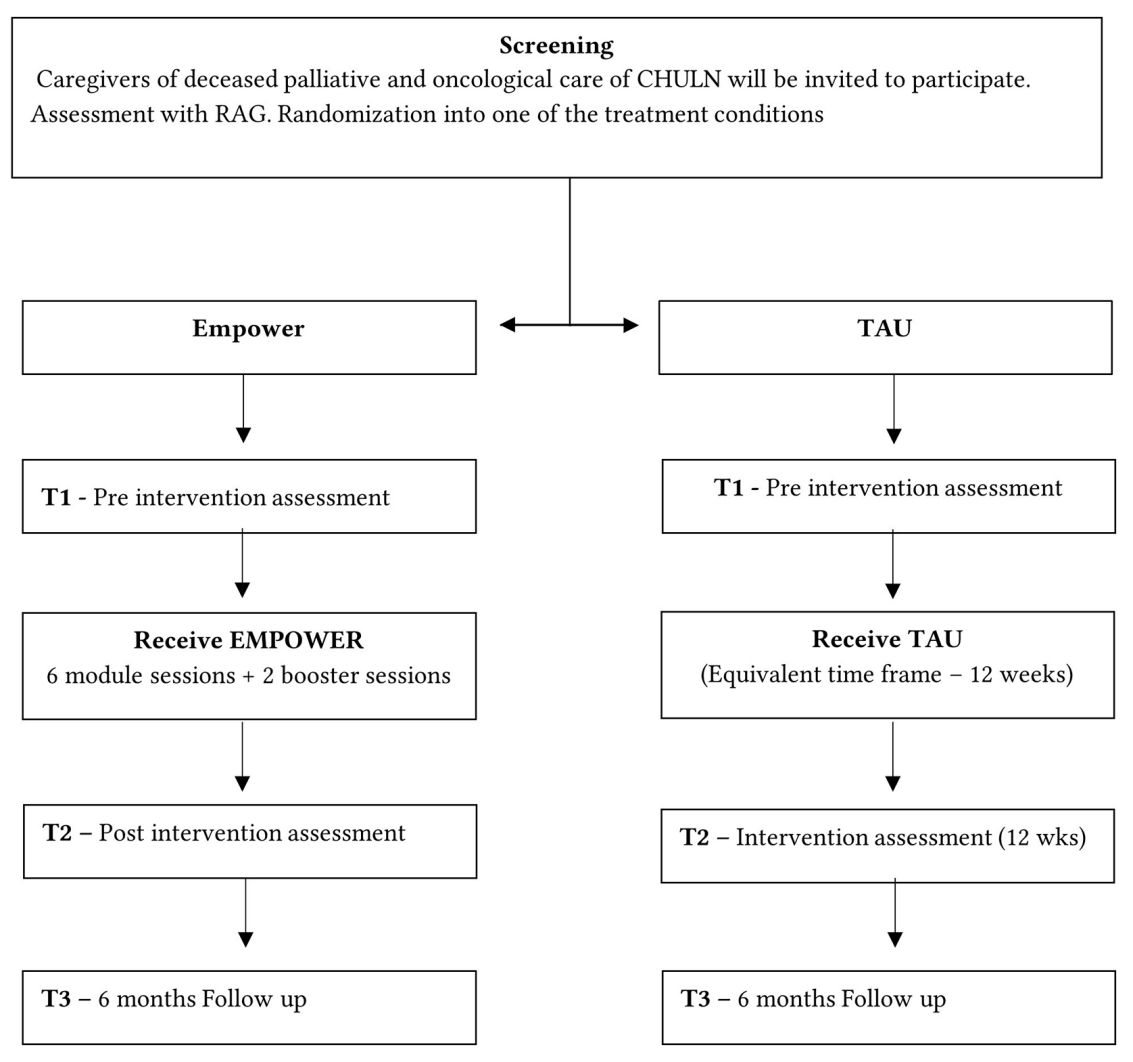
Overview of the Study Design

The present study is an exploratory Randomized Controlled Trial (RCT) with two parallel groups (Figure 1) comparing the experimental treatment conditions, Empower-Grief, and treatment as usual (TAU) in a medical centre. All study procedures have been approved by the local and central institutional review boards. The project was approved by the ethics committee of ISPA (I-138-2-24).

### Setting and Participants

Treatment is offered at CHULN Lisboa Norte to family members or other caregivers of patients, followed by the palliative care unit and oncology service. Inclusion criteria involve individuals more than 18 years old, having experienced the death of a close person (e.g., relative, partner, friend) due to cancer in a palliative or oncological context from three to 12 months, and sufficient cognitive abilities and proficiency in the Portuguese language who provide written informed consent. Participants are invited from the service registry, where the reference caregiver is identified. Exclusion criteria include individuals reporting a diagnosis of pre-existing severe or active mental disorder predating the loss (e.g., schizophrenia, bipolar disorder, major depression). Participants currently undergoing psychological intervention will also be excluded. While medication will be monitored, it will not be an exclusion criterion.

### Recruitment, Enrollment, and Randomization

Family members will be contacted by phone from the CHULN. The number of participants who refuse to participate, change their address, or are unreachable will be registered. Unreachable participants are considered those who are not reached after one month of attempts. Second, the protocol of contact is established and will be revised to address any concerns participants may have. Non-participation data will be controlled monthly (weekly at the beginning of the data-gathering period).

Participants will undergo assessment for PGD risk and then be randomly assigned to one of the two conditions. Those considered at risk, using the cut-off values (i.e., 7 or more) of Risk Assessment for Grief (Ministry of Health, 2017) to be at a moderate level, will be invited to additional intervention and participation in the current study. All participants will provide informed consent before initiating one of the two interventions. The informed consent process will cover the study's purpose, procedures, potential risks, and benefits, and it will be obtained from each participant before any data collection or randomization occurs. Participants will be informed that their data will be anonymized and securely stored.

The analysis follows an intention-to-treat principle, ensuring the inclusion of every randomized participant. Participants who do not meet the criteria will be carefully referred to an appropriate service. In cases where participants refuse, do not adhere to, or do not benefit from a particular intervention, they will be offered the intervention at a higher level in the referred services.

Randomization is independently conducted by a research assistant. Participants will be randomly assigned to Empower-Grief vs. TAU. Randomization occurs after a participant meets eligibility criteria, provides consent, and undergoes risk assessment. A random sequence of numbers or allocation codes will be generated using a randomization tool. This sequence will determine the allocation of participants to the two intervention groups. The participants will be allocated to each branch of the RCT through block randomization. Clinicians will not be aware of any research assessments, including the initial risk assessment, and will not be involved in assessing participants' outcomes.

Throughout the study, the project team will continuously monitor the randomization process to ensure it is executed as planned, with any deviations documented and addressed. The study protocol was written in accordance with the SPIRIT 2013 Statement (Standard Protocol Items: Recommendations for Interventional Trials (Chan et al., 2013).

Strategies to enhance participant retention and minimize attrition will be employed, including regular check-ins, reminders for follow-up assessments, and flexible scheduling options. Also, a feedback system for participants to express concerns or provide input on the study's procedures will be put in place, and this will be used to make necessary adjustments. Throughout their participation, we will emphasize participants’ contribution to improving grief support for others and the rigorous privacy and confidentiality measures in place.

### Treatment Conditions

#### TAU

Treatment as usual (TAU) consists of supportive psychotherapy based on a non-structured and integrative method that focuses on developing more adaptive coping strategies and understanding and working on the patient's internal models of self, others, and the world (Winston & Lujack, 2015). For the present investigation, TAU will be considered during the same period as the Empower-Grief intervention – that is, 12 weeks – as the frequency of sessions may differ slightly from case to case. Psychologists with specific training for each intervention will administer both interventions.

#### Empower-Grief

Empower-Grief (Coelho et al., 2024) is a cognitive-behavioural and acceptance-based intervention divided into six modules adapted from EMPOWER (Lichtenthal et al., 2022) to a post-mortem application. It consists of six in-presence or online 50-minute sessions and two booster sessions 2 and 4 weeks after the final intervention. It is a manualized treatment in which each session has a specific goal: 1) Welcome, initial assessment and adherence to the intervention; 2) Psychoeducation and transmission of resources for stabilization; 3) Psychoeducation on Trauma, Grief and Cognitive-Behavioral Model; 4) Promoting Experiential Acceptance; 5) Imagined dialogue; 6) Coping Training; and two final boosting sessions. In every session, the impact of the previous consultation and evolution is evaluated.

### Treatment Fidelity

Various strategies will be employed to ensure the effective delivery of the intervention and maintain consistency among psychologists. The standardized intervention protocol for "Empower-Grief" (Coelho et al., 2024) will be utilized (manualized intervention).

The implementation team for the Empower-Grief will comprise four psychologists, all with master's degrees in clinical and health psychology and at least one year of clinical experience. This group underwent a comprehensive 20/30-hour training in the Empower-Grief intervention model. In addition to the standardized training provided to all psychologists, model sessions were conducted where experienced psychologists, each with a minimum of 10 to 15 years of clinical experience in grief intervention, demonstrated how to deliver interventions effectively. These experienced psychologists will serve as valuable resources in ensuring the fidelity and quality of intervention delivery across the team.

The implementation team of TAU will include three licensed psychotherapists or MA-level psychologists in advanced postgraduate clinical training. All therapists will have at least five years of experience and at least two years of experience working with complicated grief.

During the trial, Empower-Grief psychologists will have weekly group supervision, while TAU psychologists will have their usual practices that include team reunions to discuss cases. Ongoing supervision and monitoring of psychologists in the experimental group will include regular check-ins, feedback sessions, and opportunities for providers to seek guidance or clarification. All providers will have access to the same materials, resources, and tools required for their sessions.

Fidelity checks will be conducted to ensure quality and consistency across psychologists. Purposely constructed fidelity assessment tools will include a checklist with intended tasks, content, format, and psychologists' behaviour, completed after each session and discussed in supervision. Records will be maintained for each fidelity check, including dates, psychologist names, and fidelity assessment results, along with any corrective actions taken. The primary goal of these procedures is to ensure treatment adherence, maintain low non-participation rates, and control differential non-participation rates, particularly between follow-up times and types of termination. The expected outcomes include overall low non-participation rates, specifically in the considered conditions.

### Assessments and Instruments

Three measurement points will be considered (Table 1). Considering the context of data gathering, care has been taken to avoid overburdening participants with excessive questionnaires. The screening will include sociodemographic data and RAG. The first assessment, before the first session, includes some additional sociodemographics, PG13-R, HADS, Brief COPE, ECR-RS, AAQ-II, and MSPSS. The second assessment will include PG13-R, HADS and the working alliance measure (WAI-S). The follow-up assessment period (3rd assessment) will occur six months after the last assessment. These will include all outcomes (PG13-R, HADS, PTGI). The interventions will share the same assessment periods and instruments. The follow-up assessment ensures that all participants are evaluated one year after the death, enabling a PGD diagnosis.

**Table 1:** Time Points for Measurement

Instrument	Screening	T1	T2	6 mths
Sociodemographics	x	x		
Risk Assessment for Grief (RAG)	x			
PG-13-R		x	x	x
The Hospital Anxiety and Depression Scale (HADS)		x	x	x
The Posttraumatic Growth Inventory (PTGI)				x
Brief COPE		x		
Experiences in Close Relationships (ECR-RS)		x		
Acceptance and Action Questionnaire II (AAQ-II)		x		
The Multidimensional Scale of Perceived Social Support (MSPSS)		x		
The Working Alliance Inventory-short form (WAI-S)			x	

*Note*. T1 = Pre-treatment; T2 = 12 weeks/Post-treatment; 6 months = follow up.

Participants will be contacted sensitively to inquire about the reasons for discontinuing the intervention. Clinical teams will receive supervision for each intervention, and adherence to treatment measures will be monitored.

#### Screening

Sociodemographic data, such as participants' gender, age, marital status, education, employment, and mental health and treatment history, are also collected.

##### Risk Assessment for Grief (RAG)

RAG is a hetero-assessment scale of PGD risk developed by the New Zealand Ministry of Health (Ministry of Health, 2017) and adapted for the DGS norm 003/2019 (DGS, 2019). It includes four items (anger, accusation/guilt, current relationships and general coping with grief) evaluated on a 5-point Likert scale. This instrument classifies the degree of PGD risk as low (<7 points), moderate (7-10 points) and high (≥ 10 points).

#### Primary Outcome

##### Prolonged Grief Scale – Revised (PG13-R)

The PG13-R is a self-report scale developed by Prigerson and colleagues (Prigerson et al., 2021) based on DSM-5-TR criteria for PGD. PG-13-R includes 13 items, evaluated on a 5-point Likert scale. PG-13-R grief symptoms represent a unidimensional construct with high degrees of internal consistency in research conducted at Yale (Cronbach's α = .83), Utrecht (Cronbach's α = .90), and Oxford (Cronbach's α = .93) universities. PGD diagnosis is attributed when the person obtains a total value greater than 30 and meets the temporal (12 months) and impairment criteria.

#### Secondary Outcome

##### The Hospital Anxiety and Depression Scale (HADS)

The HADS, originally developed for individuals with somatic diseases in hospitals (Zigmond & Snaith, 1983), measures anxiety and depression symptoms. The scale was developed for non-psychiatric populations and includes 14 items evaluated on a 4-point (0-3) Likert scale. Values higher than eight in each subscale suggest clinically relevant symptomatology. In the Portuguese adaptation (Pais-Ribeiro et al., 2007), Cronbach alpha values were .76 in anxiety and .81 in depression.

#### Predictors and Other Relevant Variables

##### Posttraumatic Growth Inventory (PTGI)

The PTGI (Tedeschi & Calhoun, 1996) evaluates the extent of perceived positive transformations following a challenging event. Comprising 21 items, it encompasses five subscales: personal strengths, appreciation of life, new possibilities, spiritual changes, and relation to others. Participants were instructed to assess each item on a 6-point Likert scale, ranging from 1 to 6. The overall PTGI score represents the sum of all items and higher scores indicated higher levels of posttraumatic growth. The original version of the PTGI has shown excellent internal reliability (Cronbach's α = 0.90) and acceptable test-retest reliability (*r* = .71). The PTGI has also shown good psychometric properties in breast cancer Portuguese samples (Silva et al., 2009).

##### Brief COPE

The Brief COPE (Carver, 1997) is composed of 28 items organized into 14 coping strategies. Each coping strategy is measured using two items (humour, positive reframing, emotional and social support, acceptance, religion, instrumental support, planning, active coping, behavioural disengagement, self-blaming, substance use, venting, self-distraction, and denial). Participants were asked to answer each item with a Likert-type response scale ranging from 0 (I never do this) to 3 (I always do this). Mean scores were calculated for each coping factor. The study of internal consistency for each factor using Cronbach's alpha shows adequate values (in general α ≤ .60), taking into account there are only two items per factor for the original (Carver, 1997) and the Portuguese version (Ribeiro & Rodrigues, 2004).

##### Experiences in Close Relationships (ECR-RS)

The ECR-RS (Fraley et al., 2011) is a self-report instrument that measures adult attachment to relevant persons – in this case, the deceased – rated on a seven-point Likert scale that ranges from 1 (strongly disagree) to 7 (strongly agree). Its nine items are grouped into two dimensions: attachment-related anxiety (Items 1-6) and avoidance (Items 7-9). The total subscale score consists of the mean of the items and ranges from 1 to 7, with higher scores indicating higher attachment avoidance or anxiety. The Portuguese version showed adequate reliability (α ranged from .72 to .91) and construct validity (Moreira et al., 2015).

##### Acceptance and Action Questionnaire II (AAQ-II)

The AAQ (Bond et al., 2011) is a 7-item questionnaire with a single-factor structure measuring acceptance, experiential avoidance, and psychological inflexibility. In the Portuguese validation (Pinto-Gouveia et al., 2012), results from a Confirmatory Factor Analysis showed the goodness of fit of the model, and this version also demonstrated an excellent level of internal consistency (α = .90) and good convergent and discriminant validity. Individuals are asked to rate each statement on a 7-point Likert scale ranging from 1 (never true) to 7 (always true). This scale reflects the single domain of psychological inflexibility, with higher scores indicating greater psychological inflexibility.

##### The Multidimensional Scale of Perceived Social Support (MSPSS)

The MSPSS (Zimet et al., 1988) measures perceived social support from family, friends, and others. The 12 items are grouped into three factors (family, friends and significant others), each with four items, using a 7-point Likert scale (0 = strongly disagree, 7 = strongly agree). Examples of items include “There is a special person who is close by when I need him/her"; "I can talk about my problems with my family”. The Portuguese version of the MSPSS (Carvalho et al., 2011) demonstrated good psychometric qualities (α ranged from .85 and .95).

##### The Working Alliance Inventory-Short Form (WAI-S)

The WAI-S (Tracey & Kokotovic, 1989) is a widely studied 12-item self-report that measures how the client perceives the therapeutic alliance with the therapist, with a 7-point Likert scale ranging from 1 (never) to 7 (always). In addition to the overall alliance score, the WAI-S includes three specific subscales: bond, tasks, and goals (four items, respectively). Internal consistency for the Portuguese version is .89 for the patient version and .85 for the therapist version (Machado & Horvath, 1999).

### Statistical Analysis

To evaluate the impact of Empower-Grief relative to TAU on the primary outcome (prolonged grief symptoms) and secondary outcome (anxiety and depression), we will use a mixed-effects model. A mixed-effects model for longitudinal data (pre-post and a 6-month follow-up) accounts for the hierarchical structure of the data, with participants nested within treatment groups and the correlation between repeated measurements within subjects. The intention-to-treat principle will be followed in the analysis, ensuring that all participants randomized to each treatment condition are included in the analysis, regardless of whether they completed all assessments or adhered to the treatment protocol. To address missing data, multiple imputations will be employed, a statistical method that generates multiple plausible values for missing data points based on the observed data. This method preserves the relationships between variables and reduces the potential for bias due to missing data.

Before drawing inferences, a preliminary examination of model assumptions, including checks for the normality of residuals and homogeneity of variances, will be conducted. The primary outcome, prolonged grief severity, will be modelled using a linear mixed-effects model with fixed effects for treatment condition (Empower-Grief vs. TAU), time point (pre-treatment, post-treatment, and 6-month follow-up), and their interaction, and random effects for participants and treatment groups. Secondary outcomes, anxiety and depression symptoms, will be analyzed using similar mixed-effects models.

The sample size for this research project was estimated using G*Power software, employing a repeated measures analysis with a mixed-effects model. Assuming a low effect size of *d* = 0.25, a significance level of α = 0.05, and a power of 1-β = 0.80. With two treatment groups, Empower-Grief and treatment as usual, and measurements taken at three moments (baseline, post-treatment, and 6-month follow-up), the resulting estimated sample size for the study was 44 participants for both treatments, representing a robust size to detect the anticipated effect with high statistical power.

To assess the predictive value of the considered predictors (i.e., posttraumatic growth, coping, attachment to the deceased, psychological flexibility, social support, and therapeutic alliance) on treatment outcomes, separate regression analyses will be conducted for each outcome measure (prolonged grief, anxiety, and depression). For each outcome, we will fit a mixed-effects model with the baseline variable as a fixed effect, adjusting for treatment condition, time, and their interaction. The random effects’ structure will remain the same as in the primary analysis.

## Results

The RCT is ongoing. We have contacted 294 potential participants and recruited 44 to this moment. Recruitment will close in April 2024. We plan to complete data analyses by December 2024.

## Discussion

This study responds to a lack of research in grief interventions, which is relevant considering the high prevalence of PGD and the general delay in providing care. Assessing the differentiation of intervention leads to increases and equality in access to empirically based interventions. By empowering caregivers to respond to a challenging grief process, the intervention may contribute to preventing pathological long-term psychological reactions. The present research project aims to assess the efficacy of a low-intensity selective intervention compared with TAU in terms of prolonged grief symptoms and distress. The project's second goal is to identify potential predictors of the outcome and adherence to these treatments – considering the matching conditions.

This research has several strengths. First, the study of low-intensity interventions – recommended by international and national bodies (Shear et al., 2017) – has a good cost-benefit ratio and increases access to psychological interventions in this social context. Second, Empower-Grief is flexibly delivered so that it can be adapted to caregivers’ dynamic needs and post-mortem context. Third, it was developed and refined using stakeholder feedback to optimize its acceptability and fit for its recipients’ specific needs. Also, identifying predictors of adherence and change serves to increase sensitivity in aligning interventions to the needs of clients.

The fact that the current study is conducted in a practice setting leads to some constraints – namely, the lack of manualization of TAU or the need to keep the research questionnaire relatively small. However, the practice-based nature of the context of data gathering provides a demonstration of how a structured intervention such as Empower-Grief can be useful. Results from this study will, therefore, provide a pathway for improving clinical decision-making and tailoring treatments to an individual's specific needs, which is crucial to meeting citizens’ needs in the context of the rational provision of services.

This research is situated in the field of intervention personalization and psychological services research. It can inform and enrich the predicting process by considering other dimensions of patient characteristics, such as psychological flexibility or attachment, thus fostering new research and informing bereavement support practice.

## Trial Registration

Trial registered at ClinicalTrials.gov, Identifier: NCT06270381 (registered February 21, 2024).

## Reporting Guidelines

The present report was written in accordance with the SPIRIT 2013 Statement (Standard Protocol Items: Recommendations for Interventional Trials).

## Social Media Accounts

*David D. Neto:* LinkedIn, ResearchGate
